# Supplementary material for: Chromothripsis during telomere crisis is independent of NHEJ, and consistent with a replicative origin
Source: Genome Res. 2019 May;29(5):737–49. doi: 10.1101/gr.240705.118 (PMC6499312; doi:10.1101/gr.240705.118)
Supplement: Supplemental Material [file supp_gr.240705.118_Supplemental_file_1.zip › contigs/annotated_contigs/DB92/contig.2.DB92_length_545_mean_cov_7.70642201835.docx]

**DB92_length_545_mean_cov_7.70642201835**

```
AAGAATATACCTAACCAAGGAGGTGAAAGATCTCTACAAGGAAAATTGCAAAACACTGCTGAAAGAAATCACTGAAGACACAAACAAAT
 >chrX:97699068-97699380 + E=2e-168 p=4e-02
GGAAATACATCCCATGCTCATGGATGGGTAGAATCAATATTGTGAAAATGACCATACTGCCAAAAGCAATCTACAAATTCAATGCAATT

TCCATAAAAATACCACCATTATTCTTCACAGAACTAGAAAAAAAATCCTAAAATTCATACAGAATCAAAAAGAGCCCACATAGCCAAAG

CAAGACTAAGCAAAAAGAACACATCTGGAGGCATCACATTCCTT|TTGCAAATCAAAACCACAATTAGATACCACCTTAAGAATGACCA
                                              >chrX:97700068-97700303 + E=6e-125
TAATTTAAAAATAAAAAAAAAAAACAGATGTTGGCATGGATGTTGTGAAAAGGTAACACTTTTACACTGCTGGTAGGAATGTAAGCTAG

TACAATCACTGTGGAAAACAATATGGAGATTCCTTAAAGGACTAAAAGTAGAACTACCAGCAATCCCACTATGGGGTATCTATCCAGAG

AAAAAGTCATCG
```
